# The COVID-19 basic reproductive ratio using SEIR model for the Middle East countries and some other countries for two stages of the disease

**DOI:** 10.1186/s42269-021-00572-4

**Published:** 2021-06-12

**Authors:** Marwan Al-Raeei

**Affiliations:** Faculty of Sciences, Damascus University, Damascus, Syrian Arab Republic

**Keywords:** Reproductive ratio, COVID-19, Runge–Kutta method, SEIR model, Numerical simulation, Middle east, Epidemiology, Differential equations, Pandemic

## Abstract

**Background:**

The new coronavirus disease appeared at the end of 2019, and it is now a global problem over the world. There are multiple epidemiologic indicators used for discussing the spread of pandemics, one of these indicators is the basic reproductive ratio which indicates whether the pandemic is going to spread more or relax, and the aim of this work is estimating this ratio for the Middle East countries for two stages of the pandemic.

**Main body of the abstract:**

We employ Runge–Kutta method on SEIR model to simulate the basic reproductive ratio values of SARS-CoV-2 disease by using the recorded data of the disease for two stages, up to date May 29, 2020, in the first stage and up to date September 7, 2020, in the second stage. We estimate the coefficient of exposing rate, the coefficient of infection rate, the coefficient of recovery rate and the coefficient of mortality rate of the new coronavirus disease in addition to the basic reproductive ratio values of the disease in the Middle East countries, namely Bahrain, Cyprus, Egypt, Iran, Iraq, Jordan, Kuwait, Lebanon, Oman, Qatar, Saudi Arabia, the Syrian Arab Republic, the United Arab Emirates, Turkey and Yemen where we apply the SEIR epidemic model.

**Short conclusion:**

We find that the values of the basic reproductive ratio of the new coronavirus disease based on the used model in the Middle East countries start from 1.5583 to 3.0395 in the first stage and from 1.0001 to 4.5757. Besides, we find that the problem of the new coronavirus disease in Lebanon and in the Syrian Arab Republic is not good at all in the recent stage based on the values of the basic reproductive ratio comparing with other Middle East countries. Also, we find that the value of the basic reproductive ratio for the second stage is near one; however, if we apply the method for the following stages, we find that the values return to be larger because lots of people in that stage and after did not follow the governmental procedures for stopping the spreading of the disease.

## To the editor

### Background

The new coronavirus disease appeared in Wuhan in China at the end of 2019 and caused a very big problem over the world. A lot of tools can be used for demonstrating the spreading and the forecasting of the disease; in this work, we estimate the basic reproductive ratio of the new coronavirus disease which represents the expected cases which is generated by one infectious case in a certain population with a specific pandemic, and the basic reproductive ratio value is very important tool with any pandemic because it predicts that the disease is relaxed when the value is less than one or change to epidemic when the value is more than one. There are multiple models in epidemiology used to simulate the forecasting of a specific pandemic, some use fractional derivatives, and others use ordinary differential equations. Some models take the mortality into accounts such as the SIRD model (Susceptible cases of the pandemic individual, Infectious cases of the pandemic individual, Recovered cases of the pandemic individual and Deceased cases of the pandemic individual), and others take the vaccines into accounts such as the SIRV model (Susceptible cases of the pandemic individual, Infectious cases of the pandemic individual, Recovered cases of the pandemic individual and Vaccinated cases of the pandemic individual). The first derivation of epidemiologic models was by Kermack (1927), Kamara et al (2020) found analytical solution for post death transmission model for Ebola epidemics, Khan et al (2020) discussed the effects of underlying morbidities on the occurrence of deaths for the new coronavirus disease patients, Lifshits and Neklyudova (2020) discussed mortality rate in Russian regions for the new coronavirus disease, Adedire and Ndam (2021) applied a model of dual latency compartments for the transmission dynamics of the new coronavirus disease in Nigeria, Neto et al. (2020) discussed the effect of the new coronavirus disease with the fourth industrial revolution, Osemwinyen and Diakhaby (2015) discussed the transmission of Ebola disease with the models, Rejaur-Rahman et al (2020) applied geospatial modelling for the new coronavirus disease, Akanda and Ahmed (2020) discussed the controlling of the disease in Bangladesh regions, Roy et al (2020) discussed the spatial predication using ARIMA (autoregressive integrated moving average) model, Santosh (2020) discussed the predication models for the new coronavirus disease with unexploited data, Kadi and Khelfaoui (2020) and Lounis and Al-Raeei (2021) applied the models for the spreading of the new coronavirus disease for Algeria, Aabed and Lashin Maha (2020) applied analytical study of the factors that influence the new coronavirus disease spread, Ali et al. (2020) discussed the linkage between PM 2.5 levels and the new coronavirus disease spread and its implications for socioeconomic circles, Al-Raeei (2020a, b, 2021) found the indicators of the new coronavirus disease for different location countries over the worldwide, Bhadra et al. (2020) discussed the spreading of the new coronavirus disease with mortality in Indian regions, Fang et al. (2020) applied ARIMA model for Russian regions, Gao et al. (2007) applied SIR model with pulse vaccination and distributed time delay, Gupta et al. (2020) discussed the effects of geographical factors to the new coronavirus disease outbreak in India, Zhu et al. (2019) investigated the spreading process of the epidemics on multiplex networks by incorporating fatal properties, and Aidoo et al. (2021) discussed the effects of the weather on the spreading of the new coronavirus disease in Ghana. In this work, we use the SEIR model (Susceptible cases of the pandemic individual, Exposed cases of the pandemic individual, Infectious cases of the pandemic individual and Recovered cases of the pandemic individual) for purpose of estimating the basics reproductive ratio values of the new coronavirus disease. The SEIR model takes the exposed cases into part, and this model is composed of four differential equations. The first two equations of this model are nonlinear equations and describe the change of the susceptible cases with respect to the time and the change of the exposed cases with respect to the time, and the other equations describe the rate of the infections and the recovery with respect to the time (Al-Raeei 2018, 2021). There are limited actual analytical solutions of the epidemiology models, and therefore, we need to use the computational solution for demonstrating the forecasting of a specific epidemic using one of the models which it has no analytical solution.

In the following, we apply the previous epidemical model to estimate the basic reproductive ratios of the new coronavirus disease in Bahrain, Cyprus, Egypt, Iran, Iraq, Jordan, Kuwait, Lebanon, Oman, Qatar, Saudi Arabia, the Syrian Arab Republic, the United Arab Emirates, Turkey and Yemen in addition to China, France, Russia and the USA where we take two stages of the disease into account. In the section 2, we illustrate the principle of the method used to find the basic reproductive ratios and we illustrate the results of the method. In addition to that, we illustrated the discussion of these results in the same section, while in the last section, we illustrate conclusion of the full article.

## Main text

The differential equations of the SEIR model are given as follows (Al-Raeei 2021):

1 $$\frac{{{\text{d}}S(t)}}{{{\text{d}}t}} = - \frac{{\alpha _{2} }}{N}I(t)S(t) + \alpha _{1} [N - S(t)]$$

2 $$\frac{{{\text{d}}E(t)}}{{{\text{d}}t}} = - (\alpha _{1} + \alpha _{3} )E(t) + \frac{{\alpha _{2} }}{N}I(t)S(t)$$

3 $$\frac{{{\text{d}}I(t)}}{{{\text{d}}t}} = - (\alpha _{1} + \alpha _{4} )I(t) + \alpha _{3} E(t)$$

4 $$\frac{{{\text{d}}R(t)}}{{{\text{d}}t}} = - \alpha _{1} R(t) + \alpha _{4} I(t)$$

where *N* is the number of the total population, *α*_1_*, α*_2_*, α*_3_ and *α*_4_ are the parameter of exposing rate, the parameter of the infection rate, the parameter of recovery rate and the parameter of mortality rate, *S*(*t*) is the susceptible cases of the pandemic individual, *I*(*t*) is the infectious cases of the pandemic individual*, R*(*t*) is the recovered cases of the pandemic individual, and *E*(*t*) is the exposed cases of the pandemic individual.

We applied the Runge–Kutta numerical simulation method for the previous SEIR epidemiological model for estimating the basic reproductive ratio of the new coronavirus pandemic in the Middle East countries. In sense of the Runge–Kutta simulation method, the susceptible cases of the pandemic individual, the infectious cases of the pandemic individual, the recovered cases of the pandemic individual and the exposed cases of the pandemic individual are governed by the following four equations:

5 $$S(t_{{n + 1}} ) \approx S(t_{n} ) + \tau \sum\limits_{{m = 1}}^{{m = z}} {l^{1} _{m} k^{1} _{m} }$$

6 $$I(t_{{n + 1}} ) \approx I(t_{n} ) + \tau \sum\limits_{{m = 1}}^{{m = z}} {l^{2} _{m} k^{2} _{m} }$$

7 $$R(t_{{n + 1}} ) \approx R(t_{n} ) + \tau \sum\limits_{{m = 1}}^{{m = z}} {l^{3} _{m} k^{3} _{m} }$$

8 $$E(t_{{n + 1}} ) \approx E(t_{n} ) + \tau \sum\limits_{{m = 1}}^{{m = z}} {l^{4} _{m} k^{4} _{m} }$$

with the functions:

9 $$k^{1} _{1} = f_{1} (t_{n} ,S_{n} )$$

10 $$k^{1} _{2} = f_{1} (t_{n} + c_{2} ^{1} \tau ,S_{n} + \tau (a_{{21}} ^{1} k^{1} _{1} ))$$

11 $$\begin{aligned} k^{1} _{Z} & = f_{1} (t_{n} + c_{Z} ^{1} \tau ,S_{n} + \tau (a_{{Z1}} ^{1} k^{1} _{1} \\ & \quad + a_{{Z2}} ^{1} k^{1} _{2} + a_{{Z3}} ^{1} k^{1} _{3} + \cdots + a_{{Z,Z - 1}} ^{1} k^{1} _{{Z,Z - 1}} )) \\ \end{aligned}$$

12 $$k^{2} _{1} = f_{2} (t_{n} ,I_{n} )$$

13 $$k^{2} _{2} = f_{2} (t_{n} + c_{2} ^{2} \tau ,I_{n} + \tau (a_{{21}} ^{2} k^{2} _{1} ))$$

14 $$\begin{aligned} k^{2} _{Z} & = f_{2} (t_{n} + c_{Z} ^{2} \tau ,I_{n} + \tau (a_{{Z1}} ^{2} k^{2} _{1} \\ & \quad + a_{{Z2}} ^{2} k^{2} _{2} + a_{{Z3}} ^{2} k^{2} _{3} + \cdots + a_{{Z,Z - 1}} ^{2} k^{2} _{{Z,Z - 1}} )) \\ \end{aligned}$$

15 $$k^{3} _{1} = f_{3} (t_{n} ,R_{n} )$$

16 $$k^{3} _{2} = f_{3} (t_{n} + c_{2} ^{3} \tau ,R_{n} + \tau (a_{{21}} ^{3} k^{3} _{1} ))$$

17 $$\begin{aligned} k^{3} _{Z} & = f_{3} (t_{n} + c_{Z} ^{3} \tau ,R_{n} + \tau (a_{{Z1}} ^{3} k^{3} _{1} \\ & \quad + a_{{Z2}} ^{3} k^{3} _{2} + a_{{Z3}} ^{3} k^{3} _{3} + \cdots + a_{{Z,Z - 1}} ^{3} k^{3} _{{Z,Z - 1}} )) \\ \end{aligned}$$

18 $$k^{4} _{1} = f_{4} (t_{n} ,E_{n} )$$

19 $$k^{4} _{2} = f_{4} (t_{n} + c_{2} ^{4} \tau ,E_{n} + \tau (a_{{21}} ^{4} k^{4} _{1} ))$$

20 $$\begin{aligned} k^{4} _{Z} & = f_{4} (t_{n} + c_{Z} ^{4} \tau ,E_{n} + \tau (a_{{Z1}} ^{4} k^{4} _{1} \\ & \quad + a_{{Z2}} ^{4} k^{4} _{2} + a_{{Z3}} ^{4} k^{4} _{3} + \cdots + a_{{Z,Z - 1}} ^{4} k^{4} _{{Z,Z - 1}} )) \\ \end{aligned}$$

where *τ* is the step of the time, *S*(*t*_*n*_)*, I*(*t*_*n*_)*, R*(*t*_*n*_) and *E*(*t*_*n*_) are the susceptible cases of the pandemic individual, the infectious cases of the pandemic individual, the recovered cases of the pandemic individual and the exposed cases of the pandemic individual, respectively, at the moment *t*_*n*_, and *S*(*t*_*n*+1_)*, I*(*t*_*n*+1_)*, R*(*t*_*n*+1_) and *E*(*t*_*n*+1_) are the susceptible cases of the pandemic individual, the infectious cases of the pandemic individual, the recovered cases of the pandemic individual and the exposed cases of the pandemic individual, respectively, at the moment *t*_*n*+1._ The coefficients *l*_*m*_^*j*^ and *c*_*u*_^*j*^, appeared in the equation of the Runge–Kutta method (Eqs. 5–20), are the weights and the nodes of the function expansion, *a*_*qu*_^*j*^ are the Runge–Kutta matrix coefficients, all the previous coefficients can be found using Butcher tableau, and the weights are under the following condition:

21 $$\sum\limits_{{m = 1}}^{{m = z}} {l^{j} _{m} } = 1$$

First, based on the previous equations, we find the coefficient of exposing rate, the coefficient of infection rate, the coefficient of recovery rate and the coefficient of mortality rate of the SEIR model in case of COVID-19 by fitting the collected results of the susceptible cases of the pandemic individual, the infectious cases of the pandemic individual, the recovered cases of the pandemic individual, the exposed cases of the pandemic individual and the total cases of the new coronavirus pandemic in Bahrain, Cyprus, Egypt, Iran, Iraq, Jordan, Kuwait, Lebanon, Oman, Qatar, Saudi Arabia, the Syrian Arab Republic, the United Arab Emirates, Turkey and Yemen. After that, we calculate the basic reproductive ratios of the new coronavirus disease in the Middle East countries where we can get the basic reproductive ratio for a specific epidemic model based on multiple methods. Here, we employ the method which uses the eigenvalues of the Jacobian of the system of the differential equations of the SEIR model. We start by writing the Jacobian of the SEIR nonlinear system as follows:

22 $$J(S,E,I,R) = \left[ {\begin{array}{*{20}c} { - \frac{{\alpha _{2} }}{N}I - \alpha _{1} } & 0 & { - \frac{{\alpha _{2} }}{N}S} & 0 \\ {\frac{{\alpha _{2} }}{N}I} & { - (\alpha _{1} + \alpha _{3} )} & {\frac{{\alpha _{2} }}{N}S} & 0 \\ 0 & {\alpha _{3} } & { - (\alpha _{1} + \alpha _{4} )} & 0 \\ 0 & 0 & {\alpha _{4} } & { - \alpha _{1} } \\ \end{array} } \right]$$

By finding the eigenvalues of the Jacobian at the free equilibrium, we find that the basic reproductive ratio is written as follows:

23 $$R_{0} = \frac{{\alpha _{2} }}{{\alpha _{1} + \alpha _{3} }}\frac{{\alpha _{3} }}{{\alpha _{1} + \alpha _{4} }}$$

We used Mathematica software for finding the numerical results by using the Runge–Kutta method. We found the numerical values of the coefficient of exposing rate, the coefficient of infection rate, the coefficient of recovery rate and the coefficient of mortality rate of the new coronavirus pandemic using SEIR model and the Runge–Kutta method for Bahrain, where the first case was observed in February 2020, Cyprus, where the first case was observed in March 2020, Egypt, where the first case was observed in February 2020, Iran, where the first case was observed in February 2020, Iraq, where the first case was observed in February 2020, Jordan, where the first case was observed in March 2020, Kuwait, where the first case was observed in February 2020, Lebanon, where the first case was observed in February 2020, Oman, where the first case was observed in February 2020, Qatar, where the first case was observed in February 2020, Saudi Arabia, where the first case was observed in March 2020, the Syrian Arab Republic, where the first case was observed in March 2020, the United Arab Emirates, where the first case was observed in January 2020, Turkey, where the first case was observed in March 2020, and Yemen, where the first case was observed in April 2020. The numerical simulation was based on the recorded data of the all cases of the new coronavirus disease in each country of the Middle East countries. The initial values of the susceptible cases of the pandemic individual, the infectious cases of the pandemic individual, the recovered cases of the pandemic individual and the exposed cases of the pandemic individual for each country of the Middle East countries are taken as the first day of the recorded cases of the new coronavirus disease. Also, we used the conservation of the total population condition in each step of the calculations for each country. The estimated results of the coefficient of infection rate, the coefficient of recovery rate, the coefficient of exposing rate and the coefficient of mortality rate of the new coronavirus disease are shown in Table 1 up to the end of May 2020 which includes the coefficients in *d*^−1^ unit for the Middle East countries.

**Table 1:** The coefficient of exposed cases, the coefficient of infection, the coefficient of recovery and the coefficient of mortality of the new coronavirus disease in the Middle East countries up to end of May 2020

The country	$\alpha _{1} (d^{{ - 1}} )$	$\alpha _{2} (d^{{ - 1}} )$	$\alpha _{3} (d^{{ - 1}} )$	$\alpha _{4} (d^{{ - 1}} )$
Bahrain	$0.06 \times 10^{{ - 2}}$	$47.91 \times 10^{{ - 2}}$	$0.04 \times 10^{{ - 2}}$	$6.06 \times 10^{{ - 2}}$
Cyprus	$4.89 \times 10^{{ - 2}}$	$68.69 \times 10^{{ - 2}}$	$0.01 \times 10^{{ - 2}}$	$4.90 \times 10^{{ - 2}}$
Egypt	$0.01 \times 10^{{ - 2}}$	$22.5 \times 10^{{ - 2}}$	$0.01 \times 10^{{ - 2}}$	$2.59 \times 10^{{ - 2}}$
Iran	$2.63 \times 10^{{ - 2}}$	$90.36 \times 10^{{ - 2}}$	$0.02 \times 10^{{ - 2}}$	$23.83 \times 10^{{ - 2}}$
Iraq	$0.01 \times 10^{{ - 2}}$	$133.8 \times 10^{{ - 2}}$	$0.01 \times 10^{{ - 2}}$	$7.89 \times 10^{{ - 2}}$
Jordan	$0.01 \times 10^{{ - 2}}$	$4.17 \times 10^{{ - 2}}$	$0.01 \times 10^{{ - 2}}$	$2.04 \times 10^{{ - 2}}$
Kuwait	$12.82 \times 10^{{ - 2}}$	$399.37 \times 10^{{ - 2}}$	$0.15 \times 10^{{ - 2}}$	$8.71 \times 10^{{ - 2}}$
Lebanon	$0.01 \times 10^{{ - 2}}$	$6.52 \times 10^{{ - 2}}$	$0.01 \times 10^{{ - 2}}$	$1.11 \times 10^{{ - 2}}$
Oman	$4.12 \times 10^{{ - 2}}$	$136.48 \times 10^{{ - 2}}$	$0.40 \times 10^{{ - 2}}$	$5.02 \times 10^{{ - 2}}$
Qatar	$0.26 \times 10^{{ - 2}}$	$26.45 \times 10^{{ - 2}}$	$0.16 \times 10^{{ - 2}}$	$6.09 \times 10^{{ - 2}}$
Saudi Arabia	$0.05 \times 10^{{ - 2}}$	$45.83 \times 10^{{ - 2}}$	$0.02 \times 10^{{ - 2}}$	$7.83 \times 10^{{ - 2}}$
The Syrian Arab Republic	$0.01 \times 10^{{ - 2}}$	$13.67 \times 10^{{ - 2}}$	$0.01 \times 10^{{ - 2}}$	$0.01 \times 10^{{ - 2}}$
The United Arab Emirates	$0.01 \times 10^{{ - 2}}$	$14.09 \times 10^{{ - 2}}$	$0.02 \times 10^{{ - 2}}$	$4.80 \times 10^{{ - 2}}$
Turkey	$12.01 \times 10^{{ - 2}}$	$163.63 \times 10^{{ - 2}}$	$0.06 \times 10^{{ - 2}}$	$0.05 \times 10^{{ - 2}}$
Yemen	$0.00 \times 10^{{ - 2}}$	$21.88 \times 10^{{ - 2}}$	$0.00 \times 10^{{ - 2}}$	$0.28 \times 10^{{ - 2}}$

*α*_1_*, α*_2_*, α*_3_ and *α*_4_ are the parameter of exposing rate, the parameter of the infection rate, the parameter of recovery rate and the parameter of mortality rate

After finding the values of the coefficients of the model, we find the numerical values of the basic reproductive ratios of the new coronavirus disease in Bahrain, Cyprus, Egypt, Iran, Iraq, Jordan, Kuwait, Lebanon, Oman, Qatar, Saudi Arabia, the Syrian Arab Republic, the United Arab Emirates, Turkey and Yemen. We illustrate the results of the basic reproductive ratios of COVID-19 in Table 2 for Bahrain, Cyprus, Egypt, Iran, Iraq, Jordan, Kuwait, Lebanon, Oman, Qatar, Saudi Arabia, the Syrian Arab Republic, the United Arab Emirates, Turkey and Yemen for two stages of the disease where we take the first stage up to end of May 2020 and the other stage from May 2020 up to 7 September of the same year.

**Table 2:** The basic reproductive ratio values of the new coronavirus pandemic in the Middle East countries for two stages of the disease

The country	*R*_0_^May^	*R*_*0*_^September^
Bahrain	3.0395	1.2120
Cyprus	1.5726	1.0012
Egypt	1.6059	1.0028
Iran	2.0119	1.0121
Iraq	2.2102	1.0706
Jordan	2.1503	1.0002
Kuwait	2.1407	1.0250
Lebanon	2.3853	4.5757
Oman	2.0972	1.1304
Qatar	1.5651	1.0437
Saudi Arabia	1.5583	1.0176
The Syrian Arab Republic	2.2223	1.5242
The United Arab Emirates	1.6352	1.0104
Turkey	1.6448	1.0604
Yemen	–	1.1001

*R*_*0*_^May^,* R*_*0*_^Septemper^ are the basic reproductive ratio for the new coronavirus disease as of May and September for the considered countries

The same method is applied for other countries rather than the Middle East countries, and the other considered countries are China, France, Russia and the USA. The results of the coefficient of infection rate, the coefficient of recovery rate, the coefficient of exposing rate and the coefficient of mortality rate of the new coronavirus disease are shown in Table 3 up to the end of September 2020 for France, Russia and the USA.

**Table 3:** The coefficient of exposed cases, the coefficient of infection, the coefficient of recovery and the coefficient of mortality of the new coronavirus disease in France, Russia and the USA up to end of September 2020

The country	$\alpha _{1} (d^{{ - 1}} )$	$\alpha _{2} (d^{{ - 1}} )$	$\alpha _{3} (d^{{ - 1}} )$	$\alpha _{4} (d^{{ - 1}} )$
France	$0.85 \times 10^{{ - 2}}$	$12.85 \times 10^{{ - 2}}$	$2.52 \times 10^{{ - 2}}$	$1.17 \times 10^{{ - 2}}$
Russia	$8.79 \times 10^{{ - 2}}$	$37.52 \times 10^{{ - 2}}$	$9.00 \times 10^{{ - 2}}$	$9.38 \times 10^{{ - 2}}$
The USA	$1.71 \times 10^{{ - 2}}$	$13.15 \times 10^{{ - 2}}$	$2.92 \times 10^{{ - 2}}$	$2.28 \times 10^{{ - 2}}$

*α*_1_*, α*_2_, *α*_3_ and *α*_4_ are the parameter of exposing rate, the parameter of the infection rate, the parameter of recovery rate and the parameter of mortality rate

Besides, the basic reproductive ratio values for China, France, Russia and the USA are illustrated in Table 4 for two stages of the pandemic.

**Table 4:** The basic reproductive ratio values of the new coronavirus pandemic in China, France, Russia and the USA for two stages of the disease

The country	*R*_0_^May^	*R*_0_^September^
China	1.0007	1.1522
France	3.6851	4.7345
Russia	2.2299	1.0449
The USA	5.5657	2.0753

*R*_0_^May^,* R*_0_^September^ are the basic reproductive ratio for the new coronavirus disease as of May and September for the considered countries

Also, we apply the method for finding the predicated infected cases individual and the predicted recovered cases individual for Bahrain, Cyprus, Egypt, Iran, Iraq, Jordan, Kuwait, Lebanon, Oman, Qatar, Saudi Arabia, the Syrian Arab Republic, the United Arab Emirates, Turkey and Yemen for the first half of 2021. The results of these calculations are illustrated in Table 5.

**Table 5:** The predicated infected cases individual and the predicted recovered cases individual for the first half of 2021 for the Middle East countries

The country	*I*	*R*
Bahrain	677	46,256
Cyprus	140	1942
Egypt	20,084	116,960
Iran	4568	303,610
Iraq	19,827	220,360
Jordan	2873	16,463
Kuwait	1237	234,800
Lebanon	45	25,884
Oman	901	84,843
Qatar	6600	15,150
Saudi Arabia	1535	370,180
The Syrian Arab Republic	5132	9621
The United Arab Emirates	15,301	326,870
Turkey	73,909	2,493,600
Yemen	83	2047

*I* is the predicated infected cases individual and *R* the predicted recovered cases individual

As we see from Table 2, the value of the basic reproductive ratio of the new coronavirus pandemic in the first stage of the new coronavirus disease for Bahrain is the greatest value in the Middle East countries and the value of the basic reproductive ratio of the new coronavirus pandemic for Saudi Arabia is the smallest value between the Middle East countries which returns to the high numbers of the infectious cases in Bahrain comparing with the population of Bahrain in the first stage. Also, the values of the basic reproductive ratio of the new coronavirus pandemic are in the range [1.5–3.1] for the Middle East countries in the first stage except for Yemen where the value in this stage was very high value because there are a few recoveries. If we see the basic reproductive ratios of the second stage of the new coronavirus disease, in the second column of Table 2, we see that the biggest values of the basic reproductive ratio are for Lebanon and the Syrian Arab Republic which means that the disease in these two countries is not in good case of the ending of the pandemic. Besides, we see from Table 4 that the values of the basic reproductive ratio of the new coronavirus pandemic are in the range [1.0007–5.6000] for the other countries and the biggest value is for the USA in the first stage and for France in the second stage.

### Conclusions

We applied the SEIR model for estimating the basic reproductive ratio values of the new coronavirus disease for the Middle East countries for two stages of the disease. We used the recorded data up to date May 29, 2020, in the first stage of all cases of the new coronavirus pandemic in the Middle East countries to find the coefficient of exposing rate, the coefficient of infection rate, the coefficient of recovery rate and the coefficient of mortality rate of the new coronavirus pandemic for every country of the Middle East countries. After that, we calculated the basic reproductive ratios values of the new coronavirus pandemic for the Middle East countries for same two stages of the pandemic based on the coefficients of the SEIR model.

We found that the basic reproductive ratio values of the new coronavirus pandemic were in the range of (1.5–3.01) and (1.001–5.000) for the first and second stage of the disease, respectively, in the Middle East countries. The greatest value was found in Bahrain in the first stage, while Lebanon and the Syrian Arab Republic showed the greatest values in the second stage. Similarly, we found that the basic reproductive ratio values of the new coronavirus pandemic in China, France, Russia and the USA were in the range of (1.0007–5.6000) and (1.0500–5.000) for the first and second stage of the disease, respectively. The new coronavirus disease is the same disease in all of the countries; however, there are lots of differences between the values of the basic reproductive ratio between the different countries because each country has different governmental procedures and different responsibilities from the people. In addition, if we compare between the values of the basic reproductive ratio for the Middle East countries and for other countries, we see that the values are in the same interval approximately. We believe that the method which we applied for calculating the basic reproductive ratio values for the Middle East countries is general and can be applied for other countries such as Italy, but in this paper we applied for the Middle East countries and four other countries only.

## Data Availability

The author confirms that the data are available for non-commercial using.

## Abbreviations

SIRD: The susceptible–infected–recovered–dead
SEIR: The susceptible–exposed–infected–recovered
SIRV: The susceptible–infected–recovered–vaccinated
COVID-19: Coronavirus disease 2019
RKM: Runge–Kutta method
ARIMA: Autoregressive integrated moving average
